# Analysis of musculoskeletal side effects of oral Isotretinoin treatment: a cross-sectional study

**DOI:** 10.1186/s12891-020-03656-w

**Published:** 2020-09-25

**Authors:** Nermin Karaosmanoğlu, Cevriye Mülkoğlu

**Affiliations:** 1 Department of Dermatology, Health Sciences University Ankara Training and Research Hospital, Ankara, Turkey; 2 Department of Physical Medicine and Rehabilitation, Health Sciences University Ankara Training and Research Hospital, Ankara, Turkey

**Keywords:** Acne, Back pain, Isotretinoin, Rheumatologic disorders

## Abstract

**Background/ objectives:**

Acne vulgaris is a chronic inflammatory disease affecting the pilosebaceous unit. Isotretinoin is an effective treatment option for severe acne. The aim of this study was to evaluate musculoskeletal side effects of systemic isotretinoin treatment.

**Methods:**

Ninety-four patients with acne vulgaris and 100 sex- and age-matched controls were enrolled in this study. Only the patients who had musculoskeletal symptoms were evaluated in this study. All participants were firstly assessed by a dermatologist. The patients were asked whether they had any musculoskeletal symptoms after isotretinoin treatment, if so, the feature and duration of the symptoms were recorded. The dosage of the drug, treatment duration, incidence of arthralgia, myalgia, low back pain, sacroiliitis and tendinopathy and laboratory test results were noted. The severity of pain was assessed by visual analog scale (VAS). The severity of acne vulgaris was evaluated by Global Acne Grading Scale (GAGS). Sacroiliac radiography, magnetic resonance imaging (MRI) and rheumatologic blood tests were requested from the patients meeting Assessment of Spondyloarthritis International Society (ASAS) criteria.

**Results:**

Of the 94 patients, 71 were female and 23 were male. 47.9% of the patients had arthralgia, 53.2% had myalgia, 70.2% (66) had low back pain, 11.7% had sacroiliitis and 4.3% had tendinopathy. 37.8% of 66 patients with low back pain had inflammatory pain and 62.2% had mechanical pain. Bone marrow edema consistent with sacroiliitis was detected by sacroiliac MRI in 11 patients with inflammatory back pain. The median total cumulative dose of isotretinoin was significantly higher in patients with low back pain than in patients without low back pain (*p* = 0.014). There was no significant correlation between cumulative dose of drug, treatment duration and VAS with ESR and CRP (*p* > 0.05). Also no correlation was found between GAGS scores and musculoskeletal symptoms (*p* > 0.05).

**Conclusion:**

Low back pain is one of the very common complications of isotretinoin. It can be mostly mechanical or inflammatory. Isotretinoin-induced low back pain is dose-related, and inflammatory back pain without sacroiliitis is also frequent. The clinicians should be aware of the back pain may be a reflective of sacroiliitis during isotretinoin usage.

## Background

Acne vulgaris is a chronic inflammatory disease affecting the pilosebaceous unit with multifactorial etiology [1]. Isotretinoin is an effective treatment option for severe acne vulgaris. Isotretinoin has a wide spectrum of side effects, including multiorgan systems such as reproductive, mucocutaneous, ocular, neurological, musculoskeletal and hepatic systems. It may also cause several musculoskeletal side effects such as arthralgia, myalgia, back pain, spondyloarthropathy-related symptoms and sacroiliitis [1–3]. Arthralgia and myalgia have been reported in 2–5% of patients receiving oral isotretinoin > 0.5 mg/kg/day [4]. Musculoskeletal pains and arthralgia are the most common rheumatologic side effects of the drug that can be can be detected in 20% of the patients [1, 5]. Other uncommon musculoskeletal disorders related with isotretinoin are hyperostosis, extraspinal calcifications, enthesitis, arthritis, costochondritis, osteoporosis, growth retardation, premature epiphyseal closure in children and as well as gout [3, 6–8].

In the literature, there are only a few original studies investigating the musculoskeletal side effects of isotretinoin [1, 2, 9, 10]. Furthermore, there are many case reports or case series indicating the musculoskeletal side effects of isotretinoin [11–20]. The majority of the recently performed studies are the case studies regarding with isotretinoin-induced sacroiliitis [11, 13, 16, 18, 20]. To the best our knowledge, there is no controlled study investigating the presence of isotretinoin-related musculoskeletal side effects with a wide spectrum such as arthralgia, myalgia, low back pain, sacroiliitis, tendinopathy and enthesopathy. In previous studies, the incidence of sacroiliitis and back pain were the most detected parameters [1, 2, 10].

The primary aim of this study was to evaluate and emphasize the musculoskeletal side effects of systemic isotretinoin treatment in patients with acne vulgaris and to compare them with healthy controls. The second aim was to elucidate clinicians regarding with isotretinoin-induced musculoskeletal symptoms.

## Methods

A total of 94 patients with moderate to severe acne vulgaris treated with systemic isotretinoin and 100 sex- and age-matched controls who were admitted to Ankara Training and Research Hospital, Department of Dermatology, between September 2018 and April 2019 were enrolled in this cross-sectional study. The local ethics committee approved the study. All participants were informed about the study and their written consent form was obtained.

The patients with moderate to severe acne vulgaris who receiving no other treatment modalities, no history of low back pain, ankylosing spondylitis and spondyloarthropathy, and aged over than 18 years were included in the study. Isotretinoin group included the patients under isotretinoin treatment for acne vulgaris, but had no history of rheumatologic syndromes. The control group was selected from among health professionals who had received a routine medical checkup in the hospital.

Exclusion criteria for this study were the presence of any chronic rheumatological, dermatological diseases or any patients with a history of mechanical back pain, inflammatory back pain, sacroiliitis, enthesitis, before starting isotretinoin, history suggestive of spondyloarthropathies (reactive arthritis, ankylosing spondylitis, inflammatory bowel disease, and psoriasis), or systemic autoimmune disorders. We did not include the patients who had musculoskeletal symptoms such as low back pain before isotretinoin usage in the patient’s anamnesis. Also, the patients with depression or similar psychiatric diseases, had renal and liver function disorders, those who were pregnant or using any systemic drugs for other diseases, were not included.

All participants were firstly evaluated by a dermatologist and questioned carefully about the musculoskeletal symptoms. Only the patients who had musculoskeletal symptoms such as myalgia, arthralgia, back pain were determined and enrolled in the study. They were referred to the physical medicine and rehabilitation department and examined by a specialist. Sociodemographic information, including age, sex, history of drug use (dose and duration), history of chronic diseases were recorded. A detailed anamnesis was obtained and a careful dermatological and physical examination was performed by both of the specialists. It was queried that whether myalgia, arthralgia and low back pain occurred after starting isotretinoin treatment. Data were recorded on a standardized pre-prepared evaluation form. The pain severity of the participants was evaluated by visual analog scale (VAS) based on a chart numbered from 0 (no symptom) to 10 (maximum severity).

The severity of acne vulgaris was assessed by using Global Acne Grading Scale (GAGS). According to GAGS, the body was divided into six regions -forehead, nose, each cheek, chin and back. In each region, each type of lesion is given a number: zero for no lesion, one for comedones, two for papules, three for pustules and four for nodules. Acne severity based on the GAGS score is considered as mild (1–18), moderate (19–30), severe (31–38) and very severe (≥39).

It was investigated that whether there was a enthesitis by a detailed clinical examination. The presence of enthesitis was assessed with detecting tenderness by pressure to each enthesis until blanching of the examiner’s nail bed [21, 22]. The following entheses were examined for tenderness and swelling bilaterally: common extensor tendon insertion on the lateral epicondyle of the humerus, quadriceps tendon, patellar tendon, tibial tuberosity, knee medial collateral ligament, Achilles tendon, and plantar fascia insertion on the calcaneus [23]. The presence of spontaneous entheseal pain, entheseal pain generated by pressure and/or mobilisation and/or contraction against resistance, or local entheseal swelling was interpreted as clinically enthesitis [24]. If there was a swelling and erythema, it was considered as inflammatory enthesitis. The absence of swelling or erythema was considered to be mechanical enthesitis.

Inflammatory low back pain was evaluated by Assessment of Spondyloarthritis International Society (ASAS) criteria [25]. The ASAS criteria consist of commencement under the age of 40, insidious onset, relief with exercise, no relief with rest and nocturnal pain (improving with rising up from bed). These 4 items are essential in diagnosing inflammatory low back pain. The various imaging modalities, including conventional radiography, computed tomography (CT), magnetic resonance imaging (MRI) and bone scintigraphy are used for investigation of inflammatory changes at the sacroiliac joints. In early and acute stages of sacroiliitis the diagnosis can be difficult because conventional radiographs may be normal. Inflammatory back pain is not a specific indicator of sacroiliitis. Therefore, there is need for valuable imaging methods. Scintigraphy lacks specificity. CT is a very good method for visualization of established bony destruction or ossification. MRI can identify both inflammation and structural changes, localise different degrees of inflammation and bone marrow edema, and differentiate a possible septic sacroiliitis. MRI is the most sensitive and specific modality for sacroiliitis by directly imaging changes in the synovium, articular cartilage, and subchondral bone [26–28]. In our study, the patients meeting ASAS criteria for inflammatory back pain were evaluated in detail and requested both sacroiliac radiography and sacroiliac MRI. Sacroiliac MRI was performed on 1.5 T systems. At least 12 slices of coronal oblique T1-weighted turbo spin-echo and short tau inversion recovery (STIR) sequences of the sacroiliac joints were acquired. The slice thickness was 4 mm. This images were interpreted by the same reader who had received standardized training and were blinded with regard to the study groups. In addition, laboratory blood tests including rheumatoid factor (RF), erythrocyte sedimentation rate (ESR) and c-reactive protein (CRP) values were requested. Anti-nuclear antibody (ANA) was ordered to rule out other connective tissue diseases.

### Sample size calculation

G*Power sample size calculation program [29] was used to determine the sample size. The power analysis revealed that this study had 90% power using type I error (α) = 0.05, effect size = 0.5, and a two-sided t test.

### Statistical analysis

Data were analyzed using SPSS software version21.0 (IBM Corp., Armonk, NY, USA). The normality of the data was tested by Kolmogorov-Smirnov test. Categorical datawere presented as the number (percent), and continuous data were expressed as the mean ± standard deviation or as the median (interquartile range: IQR) as appropriate. For the comparison of the paired groups, the independent samples t-test (normal distribution) and the Mann-Whitney U test (non-normal distribution) were used for the quantitative data. Chi-squared test was used to evaluate whether there was a difference in terms of musculoskeletal symptoms between the study groups. The relationship between the non-normally distributed variables was analysed with Spearman’s correlation analysis and between the normally distributed variables with the Pearson’s correlation analysis. Linear regression analysis test was used to evaluate the relationships between arthralgia, myalgia, lumbalgia, sacroiliitis and tendinopathy/entesopathy complaints and age, drug dose (isotretionin), duration of use. In all tests, *p* < 0.05 was considered as statistically significant.

## Results

This case-control study included 94 (71 women, 23 men) patients with acne vulgaris receiving isotretinoin and 100 (74 women, 26 men)age- and sex-matched controls. The age and sex distributions of the study and control groups are presented in Table 1.

**Table 1:** Demographic features of the participants

	Isotretinoin group (*n* = 94)	Control group (*n* = 100)	*p value*
Gender			
FM (n/%)	71 (75.5%)	74 (74%)	0.80
M (n/%)	23 (24.5%)	26 (26%)	
Age (Mean ± SD) years	20.8 ± 4.0	21.5 ± 3.4	0.10

*FM* Female; *M* Male; *n* Number; *SD* Standard deviation. Statistical significance was set at *p* < 0.05

**Fig. 1:**
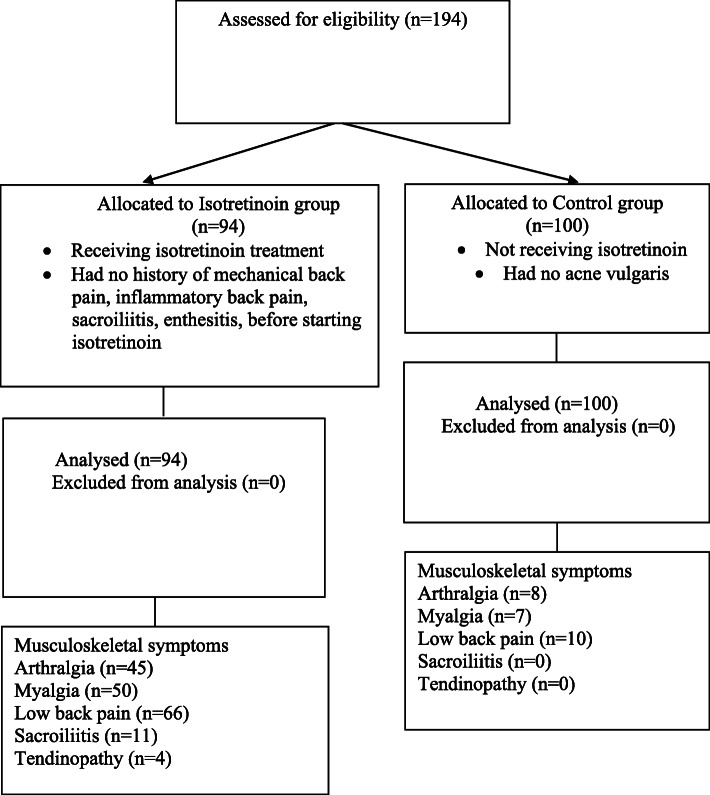
Flow chart of the participants

47.9% (45 cases) of the 94 patients had arthralgia, 53.2% (50 cases) had myalgia, 70.2% (66 cases) had low back pain, 11.7% (11 cases) had sacroiliitis and 4.3% (4 cases) had Achilles tendinopathy, in isotretinoin group. All of the tendinopathies were mechanical feature, swelling or erythema was not observed. In the control group, 8% of the subjects (8 cases) had arthralgia, 7% (7 cases) had myalgia and 10% (10 cases) had low back pain and there was no sacroiliitis and tendinopathy. There was a statistically significant difference in terms of musculoskeletal symptoms between the groups (*p* < 0.001). The flow chart of the participants was presented in Fig. 1.

39 (41.5%) of the 94 patients had severe acne vulgaris, 55 (58.5%) had moderate acne vulgaris according to the GAGS scores. The median cumulative drug dose was 2400 (IQR = 3300, min.-max: 600–8400) mg. The median duration of treatment was 3 (min.-max:1–10) months.

There was a total of 66 patients (70.2%) with low back pain in the isotretinoin group. 25 of them (37.8%) had inflammatory back pain according to ASAS criteria, while 41 of them (62.2%) had mechanical back pain. In control group, none of the patients had inflammatory back pain, however 10 patients (10%) had mechanical back pain. Median VAS score was 6 (IQR:7) in isotretinoin group and 4 (IQR:6) in control group. There was a statistically significant difference between the VAS scores in the groups (*p* = 0.03). The clinical characteristics of both isotretinoin group and healthy controls can be seen in Table 2.

**Table 2:** The clinical characteristics of the study groups

	Isotretinoin group (n:94)	Control group (n:100)	*p* value
Median cumulative dose of drug (mg)	2400 (IQR:3300)	**–**	
Median duration of the treatment (months)	3 (min.1-max.10)	**–**	
GAGS score (n/%)		**–**	
1–18 (mild)	0		
19–30 (moderate)	55 (58.5%)		
31–38 (severe)	39 (41.5%)		
Median VAS score	6 (IQR:7)	4 (IQR: 6)	**0.03**
Arthralgia (n/%)	45 (47.9%)	8 (8%)	< 0.001*
Myalgia (n/%)	50 (53.2%)	7 (7%)	< 0.001*
Low back pain (n/%)	66 (70.2%)	10 (10%)	< 0.001*
Inflammatory back pain	25 (37.8%)	0	
Mechanical back pain	41 (62.2%)	10 (10%)	
Sacroiliitis (n/%)	11 (11.7%)	0	< 0.001*
Tendinopathy (n/%)	4 (4.3%)	0	0.053

*n* Number; *VAS* Visual analog scale; *GAGS* Global acne grading scale; *IQR* Interquartile range; Bold *p* values show significance. Statistical significance was set at *p* < 0.05

The median duration of treatment was 3 (IQR: 3, min.- max: 1–7) months in patients with arthralgia (*n* = 45); 3 (IQR: 3, min.- max.: 1–8) months in patients with myalgia (*n* = 50); 3 (IQR: 3, min.- max.:1–10) months in patients with back pain; 3 (IQR: 2, min.- max.: 3–6) months in patients with sacroiliitis and 3.5 (IQR: 4, min.- max.: 1–6) months in patients with tendinopathy.

No statistically significant difference was detected in terms of total cumulative dose of isotretinoin in patients with arthralgia compared to patients without arthralgia (*p* = 0.40). There was no statistically significant difference in terms of total cumulative dose of isotretinoin in patients with myalgia compared to patients without myalgia (*p* = 0.33). Also, we found no statistically significant difference in total cumulative isotretinoin dose in patients with sacroilititis compared to the patients without sacroiliitis (*p* = 0.11) and in total cumulative dose of isotretinoin in patients with tendinopathy compared to patients without tendinopathy (*p* = 0.75).

The median total cumulative dose of isotretinoin was 2700 mg in patients with low back pain and the median total cumulative dose of isotretinoin was 1800 mg in patients without low back pain. The median total cumulative dose of isotretinoin was statistically significantly higher in patients with low back pain than in patients without low back pain (*p* = 0.014). There was no statistically significant difference between the total cumulative isotretinoin dose in patients with inflammatory back pain and mechanical back pain (*p* = 0,25). The comparison of the patients who had musculoskeletal symptoms or not in isotretinoin group regarding as total cumulative dose of drug were given in Table 3.

**Table 3:** The comparison of the patients who had musculoskeletal symptom or not in isotretinoin group in terms of total cumulative dose of isotretinoin

	Total cumulative dose of isotretinonin (mg) (median) (IQR)	p score
Arthralgia		
Yes	2280 (2400)	*p* = 0.40
No	2100 (2220)	
Myalgia		
Yes	1920 (2080)	*p* = 0.33
No	1800 (2000)	
Low back pain		
Yes	2700 (3000)	***p*** **= 0.014**
No	1800 (1960)	
Sacroiliitis		
Yes	2600 (2700)	*p* = 0.11
No	2400 (2640)	
Tendinopathy		
Yes	2100 (2190)	*p* = 0.75
No	2060 (2130)	

*IQR* Interquartile range. Bold *p* values show significance. Statistical significance was set at *p* < 0.05

In linear regression analysis, a significant and linear relationship was found between age and arthralgia, sacroiliitis and tendinopathy (*p* = ​​0.002, 0.003, 0.001, respectively). There was a significant relationship between myalgia and gender (*p* = 0.01), and it was twice as high as in women (43/71 (60.6%) versus 7/16 (30.4%), respectively). There was a significant and linear relationship between the low back pain with cumulative dose of isotretinoin (*p* = 0.02) and no significant relationship between the compared parameters with tendinopathy (*p* > 0.05). The results of linear regression analysis between the age, sex, duration of treatment and cumulative dose of drug with musculoskeletal side effects were shown in Table 4.

**Table 4:** The results of linear regression analysis between the age, sex, cumulative dose of isotretinoin, duration of treatment and musculoskeletal side effects

	Arthralgia	Myalgia	Low back pain	Sacroiliitis	Tendinopathy
Age (years)	**0.002**	0.16	0.64	**0.003**	**0.001**
Sex	0.34	**0.01**	0.93	0.61	0.24
Cumulative dose of drug	0.77	0.29	**0.02**	0.30	0.67
Duration of treatment (months)	0.98	0.62	0.09	0.59	0.94

Bold *p* values and *p* < 0.05 show statistically significance

**Fig. 2:**
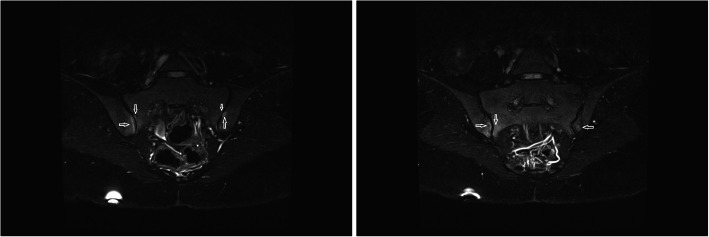
Semicoronal short tau inversion recovery (STIR) images show hyperintense lesions (arrows) consistent with bone marrow edema in bilateral sacroiliac joints

**Fig. 3:**
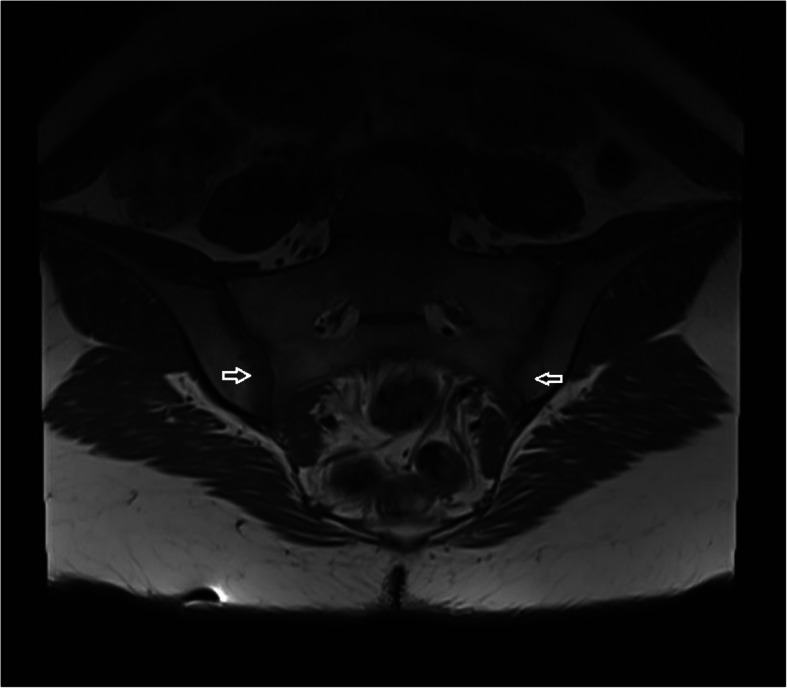
Semicoronal T1-weighted spin-echo image shows signal loss (arrows) in sacroiliac joints consistent with sacroiliitis

The mean values of CRP and ESR were 3.24 ± 1.25 mg/liter, 12.02 ± 6.55 mm/hour, respectively. None of the patients had ANA and RF positivity. The sacroiliac radiography was normal in all of the patients with inflammatory back pain. In sacroiliac MRI, sacroiliitis was observed in 11 (11.7%) patients. Nine of the 11 patients had bilateral and 2 had unilateral bone marrow edema on iliac and/or sacral wings. There was hyperintense signals on STIR images consistent with sacroiliitis (Fig. 2) and signal loss in the same location on T1-weighted spin-echo image (Fig. 3).

There was no correlation between the cumulative dose of isotretinoin, treatment duration and VAS with ESR and CRP (*p* > 0.05, for all). Also no significant relationship was found between GAGS scores with musculoskeletal symptoms (*p* > 0.05).

The patients who developed rheumatologic symptoms during isotretinoin usage were called to follow-ups by monthly. They were evaluated by a specialist and physical examination was performed at each time they came to control. VAS scores of the patients were also assessed at each follow-up. In addition, routine biochemical blood tests were acquired from all of the patients in isotretinoin group.

The patients who developed musculoskeletal symptoms such as mechanical back pain, arthralgia, myalgia or tendinopathy, except for sacroiliitis were initiated a nonsteroidal antiinflammatory drugs (NSAID) and continued a lower dose of isotretinoin. These symptoms were decreased dramatically within the first month and improved within the 3 months. In patients who were diagnosed as sacroiliitis, the drug was discontinued immediately and a NSAID was prescribed. We observed that the complaints of these patients were resolved within a month after cessation of the drug and VAS scores were also decreased dramatically. The symptoms were mostly disappeared by the third month. All the patients with sacroiliitis were completely symptom free at the sixth month of the discontinuation of the drug.

## Discussion

Isotretinoin, or 13-cis retinoic acid, is a vitamin A derivative used for severe recalcitrant acne since 1982. Although isotretinoin is a very effective drug, it may have many side effects. It is essential that the clinician should be careful about the various side effects of the drug [30].

In this study, we focused on the musculoskeletal side effects of isotretinoin. Ninety-four patients treated with isotretinoin suffering from musculoskeletal pain were included. The patients were then examined in detail. They were also compared with 100 healthy controls.

Isotretinoin has been associated with various musculoskeletal side effects in the recent literature. Some of these are original studies, while most of them are sporadic case reports [1, 2, 9, 11–20]. The onset of the musculoskeletal symptoms of the patients in these case reports was in the first few months. Consistent with the literature, the beginning of the symptoms in our patients was in the first few months with a median value of 3 months [1, 2, 9, 12–18]. Considering that the median duration of treatment was 3 months, it is important for the clinician to be on the alert in the first few months of the therapy.

Selçuk et al. evaluated 73 patients receiving isotretinoin due to moderate or severe acne vulgaris and asked all patients about their inflammatory back pain and musculoskeletal complaints. They have found myalgia in 42.5% and low back pain in 49.3% of the patients. Acute sacroiliitis was determined in 8.2% of them following a sacroiliac MRI. The authors concluded that the incidence of sacroiliitis in patients receiving isotretinoin is quite high [1]. In our study, the percentages of myalgia and sacroiliitis were similar to this study, however, the frequency of back pain was noticeably higher in isotretinoin group. Additionally, the median total cumulative dose of isotretinoin was significantly higher in patients with low back pain than in patients without low back pain (*p* = 0.01). This was one of the most important results of this study. Other musculoskeletal side effects were not found to be statistically significantly related with the total cumulative dose of isotretinoin. The patients with low back pain using isotretinoin should be asked about the dosage and duration of their drug, and if necessary, the dose should be reduced. On the other hand, it is an interesting finding that sacroiliitis is not related with the total cumulative dose of the drug.

In their study, Alkan et al. compared two acne vulgaris group receiving either isotretinoin or tetracycline. In the isotretinoin group, 23.1% of the patients were found to have spondyloarthropathy-related symptoms. No inflammatory back pain was observed in tetracycline group (32 patients). They found unilateral sacroiliitis only in one patient in the isotretinoin group that included a total of 42 patients. They also emphasized that all rheumatologic symptoms of the patients disappeared after the discontinuation of the drug and the complaints were drug related [2]. In the present study, 37.8% of the patients in the isotretinoin group with low back pain were found to have inflammatory back pain. The total cumulative dose of isotretinoin does not seem to be in relationship with mechanical or inflammatory back pain.

In another study, the effect of isotretinoin on Achilles tendinopathy was investigated. The authors divided a total of sixteen rats into two groups and administered isotretinoin for the first group and soy oil for the second group. After 6 weeks, Achilles tendons were excised and evaluated histopathologically. In the isotretinoin group, a biomechanical and histopathological negative effect on Achilles tendon was detected. Therefore, the authors recommended asking the patients with tendinopathy about their use of isotretinoin in their medical history [9]. Enthesitis is a hallmark feature of the spondyloarthropathies. Although rare, enthesitis can be seen due to isotretinoin usage. Several new MRI and ultrasound scoring systems have been used for diagnosis of enthesitis. The increased thickness of entheses and hypoechogenicity are the specific ultrasonographic findings of the enthesitis [22, 31]. In our study, the diagnosis of enthesitis was based on solely clinical examination. Only four patients (4.3%) were found to have Achilles tendinopathy in isotretinoin group while none of the patients in control group had tendinopathy. Since tendinopathy can be seen in patients receiving isotretinoin, such patients should be asked whether they use the drug or not [9].

The exact pathogenesis of sacroiliitis in isotretinoin use is unclear. Isotretinoin induces some alterations in the lysosomal membrane structure of cells due to it’s detergent-like effects. This leads to a degeneration process in the synovial cells. Retinol and retinoic acid derivatives such as isotretinoin can also stimulate MMP-2 activity and cause membrane damage in the joints [8, 12, 32, 33]. It is believed that isotretinoin treatment may render cells susceptible to mild traumas that normally would not cause injury. This theory is supported by studies of the presence of sacroiliitis in athletes treated with isotretinoin [8, 32, 34]. Weber et al. evaluated 20 healthy athletes-recreational runners (12 men and 8 women) and 22 professional ice hockey players with MRI images. MRI of the sacroiliac joints of runners was performed at baseline and 24 h after running, however in the hockey players, was performed at a single time point in the last month of the competitive season. MRI scans were examined by three readers, independently. Bone marrow edema edema and fat metaplasia were observed in both groups but these lesions did not increase after running. The authors reported that 35% of the runners and 41% of the ice hockey players met the ASAS criteria of active sacroiliac joint inflammation and the most affected region was the posterior lower ilium. They said that these MRI findings could be reflective of mechanical stress injury, vascular signals, anatomic variants, or degenerative joint disease [34].

The limited extent bone marrow edema of sacroiliac joints is quite unspecific and can occur in various conditions, not related to axial spondyloarthropathy. Winter et al. reported that sacroiliitis on MRI according to the ASAS definition was found in postpartum women, healthy individuals, and runners as 57, 23, and 13%, respectively. The only one MRI finding that differentiated the HLA B27+ axial spondyloarthropathy patients from asymptomatic individuals, runners, and women with postpartum back pain was a deeper (extensive) bone marrow edema [35]. In our study, all of the patients were young and physically active. Bone marrow edema consistent with sacroiliitis was observed in 11.7% of our patients. It may be an overdiagnosis of sacroiliitis, for this reason, this results should be interpreted with caution. Our patients were symptomatic after using isotretinoin which suggest the hypothesis of drug related sacroiliitis, in addition, MRI follow up to confirm resolution is recommended.

Taheri et al. conducted a study in military personel to evaluate whether trauma or more physical activity can affect the incidence of sacroiliitis. In this cross-sectional study, 113 military personnel who receiving isotretinoin at least 3 months for acne vulgaris were investigated. The authors reported that 46.9% of the patients had low back pain. Of the patients with back pain, 54.7% (29 cases) had inflammatory back pain and 45.3% (24 cases) had mechanical back pain. Sacroiliitis was determined only in 5 patients [10]. This study have more patients but no control group. In addition, they assessed only the incidence of low back pain and sacroiliitis but not arthralgia, myalgia, tendinopathy or enthesopathy. Similarly to our study, inflammatory back pain without sacroiliitis was found in the majority of the patients. It can be claimed that the drug may cause inflammatory back pain without sacroiliitis in healthy subjects. However in our study, numbers of the patients with mechanical back pain were more higher than inflammatory back pain. We showed that mechanical low back pain is a common complication in patients receiving isotretinoin. We recommend that young patients with back pain be questioned about their drug usage, and clinicians need to be aware about this complaint may result from isotretinoin.

In healthy control group, only a few of the volunteers had musculoskeletal pain symptoms such as myalgia, arhtralgia and back pain. None of them had sacroiliitis or tendinopathy. The statistically significant high prevalence of musculoskeletal findings in drug users in the same age group was considered as an important indicator that current symptoms were significantly drug dependent.

This study has some limitations. There is a limited number of patients in total, so the results of the study should be interpreted carefully. Another limitation was that the MRI images of the patients with sacroiliitis were not scored. Furthermore, MRI follow up may be recommended for sacroiliitis positive patients to confirm resolution after drug cessation. Enthesitis was not evaluated and scored by an imaging method such as ultrasonography or MRI in our study. The diagnosis of enthesitis was depended on solely clinical evaluation. Further randomized controlled studies are needed with wider patient groups in order to elucidate the musculoskeletal side effects of the isotretinoin.

## Conclusion

Low back pain is a very common side effect of isotretinoin and it is dose-related. Although sacroiliitis is a rare complication of isotretinoin, inflammatory back pain without sacroiliitis can be seen frequently. The patients suffering from back pain should be asked for drug usage. Considering that musculoskeletal complaints are mostly temporary, they can be often overlooked by clinicians. For this reason, we recommend the clinicians to perform a more careful and close follow-up in order to carry out the most proper approach to these patients.

## Data Availability

The datasets used and/or analyzed during the current study are available from the corresponding author on reasonable request.

## Abbreviations

NSAID: Non-steroidal anti-inflammatory drug
MRI: Magnetic resonance imaging
CT: Computed tomography
ASAS: Assessment of spondyloarthritis international society
VAS: Visual analog scale
GAGS: Global acne grading scale
HLA: Human leukocyte antigen
CRP: C-reactive protein
ESR: Erytrocyte sedimentation rate
RF: Rheumatoid factor
ANA: Anti-nuclear antibody
